# Extreme cavity expansion in soft solids: Damage without fracture

**DOI:** 10.1126/sciadv.aaz0418

**Published:** 2020-03-27

**Authors:** Jin Young Kim, Zezhou Liu, Byung Mook Weon, Tal Cohen, Chung-Yuen Hui, Eric R. Dufresne, Robert W. Style

**Affiliations:** 1 School of Advanced Materials Science and Engineering, SKKU Advanced Institute of Nanotechnology (SAINT), Sungkyunkwan University, Suwon 16419, South Korea.; 2 Department of Mechanical and Aerospace Engineering, Field of Theoretical and Applied Mechanics, Cornell University, Ithaca, NY 14853, USA.; 3 Massachusetts Institute of Technology, Cambridge, MA 02139, USA.; 4 Department of Materials, ETH Zürich, Zürich 8093, Switzerland.

## Abstract

Cavitation is a common damage mechanism in soft solids. Here, we study this using a phase separation technique in stretched, elastic solids to controllably nucleate and grow small cavities by several orders of magnitude. The ability to make stable cavities of different sizes, as well as the huge range of accessible strains, allows us to systematically study the early stages of cavity expansion. Cavities grow in a scale-free manner, accompanied by irreversible bond breakage that is distributed around the growing cavity rather than being localized to a crack tip. Furthermore, cavities appear to grow at constant driving pressure. This has strong analogies with the plasticity that occurs surrounding a growing void in ductile metals. In particular, we find that, although elastomers are normally considered as brittle materials, small-scale cavity expansion is more like a ductile process. Our results have broad implications for understanding and controlling failure in soft solids.

## Teaser

Cavities in soft materials grow like voids in ductile metals.

## INTRODUCTION

Cavitation plays a key role in the failure of solids. This has long been appreciated in ductile metals, where void/cavity nucleation, growth, and coalescence govern the initiation of fracture and fatigue [e.g., (*1*–*4*)]. Thus, there is an extensive body of literature devoted to the topic [e.g., (*5*–*7*)]. Cavitation also occurs in highly elastic materials, such as rubber (*8*–*10*). In these systems, cavitation underpins processes ranging from fracture and the failure of adhesives (*8*, *10*, *11*) to traumatic brain injury (*12*, *13*). Furthermore, cavitation by the injection of fluid is emerging as a method to characterize soft materials (*3*, *14*, *15*).

However, understanding soft-solid cavitation has not been a simple question of extending results from the ductile metal literature. Researchers have typically treated cavitation in soft solids and ductile metals as separate problems, as these materials have very different properties. Metals are orders of magnitude stiffer than elastomers and gels. Ductile metals yield plastically at low strains, while soft solids can often stretch elastically to many times their original length before irreversible bond breakage occurs. Furthermore, metals are ductile, while elastomers are generally considered as being brittle.

Thus, while void growth in metals is well understood, there is still a lack of consensus on the mechanisms governing soft-solid cavitation [e.g., (*16*–*19*)]. As a singular event in space and time, cavitation pushes theory and experiment to their limits. Theoretical challenges arise primarily from the enormous deformations around the expanding cavity, which lead, among other difficulties, to a lack of valid, reliable constitutive models. Experimental challenges revolve around the fact that cavity growth typically occurs unstably (i.e., fast) and at very small scales. Thus, it is difficult to achieve sufficient spatial and temporal resolution to resolve cavity inflation and the separation of elastic, inelastic, and viscous contributions.

Here, we resolve some of these experimental difficulties by condensing liquid droplets in soft materials (*20*). This approach allows us to slowly and systematically grow and shrink liquid-filled cavities inside unfilled elastomers, without initial defects due to injection. Breaking symmetry with a macroscopic strain, we are able to easily visualize growth-induced damage. We find that small-scale cavity growth has much more in common with ductile metal cavitation than expected. In particular, at these scales, cavity growth in soft solids is rather similar to a ductile process, as bond breakage (stress softening) is distributed around the surface of the cavity, instead of being localized to a well-defined crack tip. This has important implications for understanding and controlling failure in soft solids.

## RESULTS

### Volume-controlled cavity growth

**Fig. 1:**
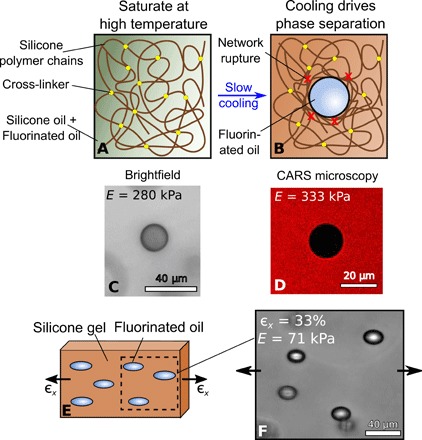
Droplet growth via phase separation in silicone gels. (**A** and **B**) Schematics showing how droplets are formed. Silicone gels are submerged at 40°C in fluorinated oil for several hours, until the gels are completely saturated. Upon slow cooling to room temperature, droplets appear. (**C**) A droplet growing in unstretched silicone with *E*= 280 kPa. (**D**) A similar droplet in silicone with *E* = 333 kPa, imaged with CARS microscopy, which visualizes the presence of the silicone network. This is clearly excluded from the growing droplet. (**E** and **F**) When a silicone gel is held with a constant, uniaxial stretch during the phase separation process, droplets grow as spheroids.

We nucleated and grew liquid inclusions in silicone gels using the technique shown schematically in Fig. 1(A and B) (*20*). We created silicone gel samples by mixing silicone polymer chains with different amounts of cross-linker to produce gels with a range of Young’s moduli from *E* = 71 − 800 kPa. The resulting gels are highly elastic, showing no evidence of a Mullins effect up to the point of failure in tensile tests consisting of repeated loading/unloading cycles of increasing amplitude (see the Supplementary Materials). The gels were immersed in a fluorinated oil (Fluorinert FC-770, Fluorochem) that is partially soluble [ ∼ 3 volume % at room temperature (*20*)] in silicone and then incubated at 40°C for several hours to allow sample saturation. Upon slow cooling to room temperature (23°C), phase separation occurs, causing nucleation and growth of fluorinated-oil droplets within the silicone gel over a time scale of tens of minutes (e.g., Fig. 1C). By controlling temperature, we effectively have direct control of droplet volume. Depending on various parameters (chiefly *E* and the cooling rate), the droplets can grow as large as several tens of micrometers in radius (*20*). They are then stable until diffusion of the oil out of the edges of the sample eventually causes them to shrink and disappear. Note that *E* will change slightly during cooling, as described by rubber elasticity theory, but this will be at most a few percent in the range of temperatures that we use.

We confirm that the polymer network is rejected from the droplets with coherent anti–Stokes Raman scattering (CARS) microscopy at a wave number of 2912 cm^−1^ (Fig. 1D). This is a spectroscopic, confocal technique that can detect the vibrational signature of silicone. The figure shows a typical, fully grown droplet, displaying the lack of silicone signal inside the droplet. We find no significant difference between the intensity in such droplets and in pure fluorinated oil, suggesting that the network is fully excluded (see the Supplementary Materials).

### Self-similar, spheroidal droplet growth

Droplets grown in stress-free gels are always observed to be spherical [see Fig. 1 (C and D) and (*20*)]. However, if we prestretch the sample with a uniaxial strain ϵ*_x_*, and this stretch is held constant during the entire incubation, nucleation, and growth process, spheroidal droplets form with their long axis parallel to the stretch direction (Fig. 1, E and F). As described below, this symmetry breaking gives us information about how damage occurs.

**Fig. 2:**
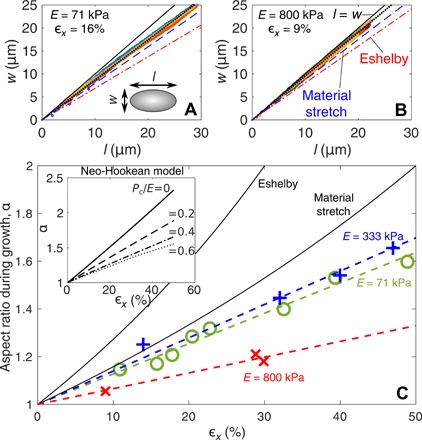
Droplet shape evolution during growth. (**A** and **B**) Droplet growth in different stiffness samples with different applied strains all grow in a scale-free way. Different colors correspond to different droplets. (**C**) The aspect ratio of growing droplets increases linearly with stretch. Dashed lines are lines of best fit, assuming that α(ϵ*_x_* = 0) = 1 (*20*). For all plots, we include the predicted shape evolution of droplets from Eshelby theory (*21*) and for a volume of solid material that stretches with the surrounding solid. Inset: The aspect ratio of inflated spherical inclusions in an incompressible, neo-Hookean solid that is stretched with strain ϵ*_x_*. Different lines correspond to different inflation pressures, *P*_c_/*E*.

For all our experiments, droplets grow with a fixed, spheroidal shape. Figure 2(A and B) demonstrates the shape evolution of droplets (length, *l*, and width, *w*, during growth) in experiments with different *E* and ϵ*_x_*. All of the droplets maintain the same aspect ratio, α = *l*/*w*, as they grow (the see the Supplementary Materials for more examples).

We see that there is a strong, linear correlation between α and ϵ*_x_*, with highly elongated droplets forming in the most stretched samples (Fig. 2C). The aspect ratio also varies with stiffness: Droplets growing in the stiffest sample, *E* = 800 kPa, remain much more spherical than droplets in the two softer samples at the same stretch. There is a nonmonotonic dependence of α on *E*, which suggests that the shape of the droplets is controlled by material parameters beyond *E* (i.e., either nonlinear elastic or failure properties).

### The pressure for droplet growth

We gain useful insight by comparing measured values of α to simple elasticity theory. For example, Eshelby’s inclusion theory (*21*) predicts that an initially spherical, incompressible, liquid inclusion, embedded in a linear-elastic solid, will deform as α = (6 + 10ϵ*_x_*)/(6 − 5ϵ*_x_*). However, it markedly overpredicts measured values (Fig. 2). Droplets actually appear “stiffer” than the silicone gel: If we take a uniform piece of material and apply a uniaxial stretch, then its aspect ratio will change to α = (1 + ϵ*_x_*)/(1 − ϵ*_x_*/2). However, the measured value of α is even smaller than this (Fig. 2). One explanation is that there is a significant surface tension, ϒ, of the droplet interface. However, we expect this to be negligible, as solid capillarity should only play a role when *w*, *l* ≲ ϒ/*E* (*22*, *23*). We estimate ϒ = 4.4 mN/m by using the surface tension of uncured polymer chains against the fluorinated oil, as measured with the pendant-droplet method [e.g., (*24*)]. This gives a value of ϒ/*E* that is much smaller than all the droplets observed (see Table 1).

**Table 1:** Measured material properties for the different stiffness silicone gels. ϒ is taken as 4.4mN/m, the surface tension of uncured silicone against fluorinated oil.

**Young’s** **modulus *E*** **(kPa)**	**Fracture** **energy Γ** **(J/m^2^)**	**Elasto-** **adhesive** **length Γ/*E*** **(μm)**	**Elasto-** **capillary** **length ϒ/*E*** **(nm)**
71	21	300	62
333	34	102	13
800	58	73	5.5

These simple expressions fail because the elastic network resists droplet growth. A significant pressure, *P*_c_, inside the droplet drives growth, accompanied by large nonlinear deformations. If this isotropic stress is large in comparison to anisotropic stresses from the macroscopically applied strain, the droplet shape will remain relatively spherical. We can demonstrate this effect with a simplified model. Consider an initially spherical hole in a stretched, nonlinear elastic solid, with far-field strain ϵ*_x_* (Fig. 2D). We inflate the hole with a pressure *P*_c_ and measure the resulting shape. For simplicity, we take the solid to be an incompressible, neo-Hookean material with small-strain elastic modulus *E* (see the Supplementary Materials for details of the solution procedure).

The simplified model captures many features of the experiments. (see Fig. 2C and the Supplementary Materials). In particular, it demonstrates how increasing *P*_c_/*E* results in rounder droplets, with aspect ratios comparable to our measured results. Furthermore, a comparison with the data suggests that the value of *P*_c_/*E* in droplets is different for the various samples, with the stiffest sample having the highest relative pressures. However, although it is useful for qualitative insight, it should not be used for quantitative comparison. Because of the well-known cavitation instability (*7*), there are no stable solutions that match the α versus ϵ*_x_* data for the sample with *E* = 800 kPa. Thus, the model ignores some key physics—especially damage to the polymer network during growth. Damage is expected, as the polymer mesh size of the gel is *O*(10nm) [e.g., (*25*)], so cavities enlarge by a few orders of magnitude during growth. Thus, the resulting strains far exceed the failure strains that are observed in macroscopic tensile tests [e.g., (*26*)].

### Damage during droplet growth

**Fig. 3:**
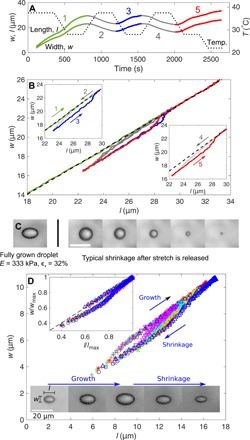
Irreversible droplet growth and shrinkage indicates bond breakage. (**A**) The programmed stage temperature (dotted track) and droplet length and width versus time for a typical droplet in a gel with *E* = 333 kPa and ϵ*_x_* ≈ 20%. No droplets nucleate or disappear during the whole cycle. (**B**) The same data plotted with *l* versus *w*. The black, dashed line shows constant aspect ratio growth. Insets show the same data, split apart to highlight the cycling behavior. (**C**) Left: A fully grown droplet in a stretched silicone. Right: The sample is cut to remove the stretch, and a droplet is imaged as it shrinks. Scale bar, 20 μm. (**D**) *l* and *w* for a selection of droplets as they grow and shrink in silicone with *E* = 333 kPa and ϵ*_x_* = 60%. Shrinkage is driven by evaporation of fluorinated oil from the sample sides. Different colors correspond to different droplets. Inset: When *l* and *w* are rescaled by the maximum size that a droplet grows to (*l*_max_ and *w*_max_), all tracks collapse onto a single hysteresis curve. The images show how a typical droplet grows and shrinks.

We rule out purely elastic growth by examining the irreversibility of droplet growth and shrinkage in a stretched sample. As a first test, we apply a temperature cycle to a stretched sample (ϵ*_x_* ≈ 20%) in a thermal stage (Instec TSA12Gi). This causes both *l* and *w* to cycle with time, as shown in Fig. 3A. Plotting *l* versus *w* (Fig. 3B) then immediately shows evidence of irreversibility: During initial growth, droplets grow in a self-similar way. However, if we then shrink the droplets and regrow them, the shape of the droplet during regrowth is more elongated (see also images in Fig. 3D). If we continue to grow the droplet larger than the size it previously attained, then it returns to the same, constant-aspect-ratio growth line that it initially grew along. This shows that the network undergoes damage between initial and subsequent growth, although our silicone gels display no sign of inelasticity before failure in macroscopic tests. Apparently, the damaged network leads to a lower pressure in the droplet upon regrowth, and this results in a higher aspect ratio (see inset of Fig. 2C).

Although droplets grow by a damage mechanism, they differ from brittle fracture in that bond breakage appears to be distributed around their surface rather than being localized to a crack tip. Figure 3C shows a typical, fully grown droplet in a stretched sample. After droplets have finished growing, the sample is cut to release stress, and we observe droplet shrinkage, as shown in the subsequent images. During this shrinkage, the droplet remains approximately spherical. This is inconsistent with localized damage, as in that case, we would instead expect the droplet to close with a lenticular, crack-like shape. Instead, damage appears to be distributed much as it would be in a ductile material. Unfortunately, we are unable to directly determine the form of this damage—for example, microcrack formation or distributed chain breakage. However, we believe that is likely due to the latter, as this is known to occur in unfilled gels at large strains (*27*).

**Fig. 4:**
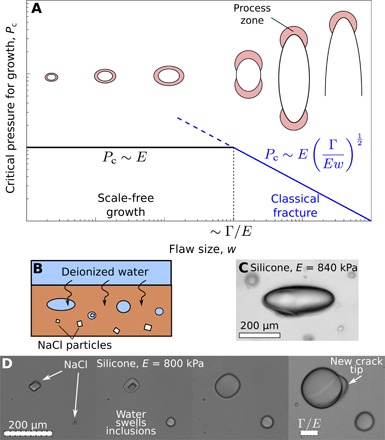
Transition from scale-free to crack-like growth. (**A**) The suggested dependence of critical pressure for flaw growth, *P*_c_ on flaw size, *w*. For small flaws (black), there is a scale-free regime with *P*_c_ constant. Large flaws (blue) behave like classical cracks. We hypothesize that the cross-over between scale-free and crack-like regimes should occur when the process zone size (∼Γ/*E* for large cracks) is comparable to the size of the flaw. The schematic figures illustrate the anticipated change in cavity and process zone morphology during growth. Note that we expect the flaw to change from aligned with to perpendicular to the stretch direction when it reaches the crack-like regime. (**B**) A schematic of the modified swelling technique for growth large inclusions. The system is always kept at room temperature. (**C**) A lenticular crack in an unstretched, stiff silicone gel formed by swelling a salt crystal. (**D**) Inclusions of different initial sizes growing in silicone, imaged at four time points. In the last frame, the largest inclusion develops a crack tip, illustrating how the transition to fracture occurs.

We can infer further information about how the damaged zone around droplets grows by comparing growth and shrinkage curves for different sized droplets. Figure 3D shows the typical evolution of the shapes of various droplets that grow and shrink (due to slow diffusion out of the side of the sample) in silicone with *E* = 333 kPa and ϵ*_x_* = 60% (c.f. supplementary movies). If we scale droplets’ growth trajectory by their dimensions at their maximum size, *w*_max_ and *l*_max_, all of the data collapse onto a single trajectory, as shown in the inset. This collapse shows that the whole growth process is self-similar and thus that the process zone must grow with the droplet, as shown schematically in Fig. 4A.

## DISCUSSION

### Cavity growth is independent of the fracture energy

The experiments show several key features: (i) cavities grow as smooth-walled spheroids, (ii) they grow in a self-similar manner, and (iii) growth is accompanied by damage that is distributed around the cavity surface rather than being localized to a crack tip. These are all at odds with crack-like growth.

We can rule out a dependence of cavity growth on the silicone’s brittle fracture properties with a simple dimensional argument. From above, α during initial growth is independent of the droplet’s size. Thus it only depends on ϵ*_x_* and the silicone’s material properties describing elasticity (e.g., *E*), fracture (the fracture energy, Γ), and damage (e.g., the stress at which inelasticity sets in, σ*_i_*)

$$\alpha =f(\epsilon_{x},\Gamma ,E,\sigma_{i}\ldots )$$ (1)

Γ has units of pressure × length, while the other material properties are either dimensionless or have units of pressure. Thus, there is no dimensionally consistent way that α can depend on Γ, so growth must be independent of Γ. This is intuitive as Γ describes the fracture process where damage is localized to a crack tip—which does not occur here.

### Growth at constant pressure *∼E*

As well as showing that growth is independent of Γ, self-similar, nonbrittle growth also shows that *P*_c_ is constant during growth. This matches previous theoretical results from the ductile void-growth literature, which showed that self-similar void growth occurs in elastic-plastic materials at constant driving stress [e.g., (*1*, *28*, *29*)]. This also explains the stability of droplets in our experiments. If *P*_c_ changes as droplets grow, then we would expect transport of fluorinated oil between droplets of different sizes—i.e., ripening—even when the temperature is held constant. However, in recent work, we observed no evidence of this (*20*).

The magnitude of the constant growth pressure is *O*(*E*). This has been shown in multiple cavitation experiments [e.g., (*14*, *17*)] and is supported by a comparison between the model and data in Fig. 2C. However, the exact value of *P*_c_ will be determined by the constitutive relationship of the material at very large deformations, including inelastic and nonlinear elastic contributions. In particular, the fact that *P*_c_ depends on damage to the material could explain why cavitation experiments in soft gels do not always give good agreement with the long-established limit for elastic cavitation in incompressible neo-Hookean solids: *P*_c_/*E* = 5/6 (*8*, *9*, *14*, *15*, *17*).

### Different regimes of cavity growth behavior

Our results suggest that there are different regimes of pressure required to open a flaw in a gel/elastomer, as shown in Fig. 4A. For small cavities, growth is scale free, with *P*_c_ ∼ *E* constant. For large cavities, growth is known to be crack-like, with damage localizing to a crack tip (*15*, *18*). Then, linear elastic fracture mechanics gives that $P_{c}∼\sqrt{\Gamma E/w}$ [e.g., (*30*)].

We can naïvely predict the transition point between these two regimes by equating the two expressions for *P*_c_ to find a crossover at *w* ∼ Γ/*E* (see Fig. 4A). This explains why we never see fracture in our phase-separation experiments. In Table 1, we report measured values of Γ/*E*. For all the experiments, *w* ≪ Γ/*E*, so we expect self-similar growth.

To explore the transition to fracture, we use a modified technique, shown schematically in Fig. 4B. We cure small, sodium chloride crystals inside the silicone (salt is insoluble in silicone and does not alter the cross-linking process) (*31*). Subsequently, we immerse the unstretched silicone in deionized water. This diffuses through the silicone, dissolves the salt, and swells the inclusions (Fig. 4, C and D). Initially, all inclusions swell, becoming more spherical. However, larger inclusions suddenly develop a crack tip and subsequently grow to form lenticular cracks, as expected in the fracture regime.

These results are consistent with the behavior predicted above. For example, in Fig. 4D, inclusions grow in a silicone gel with Γ/*E* = 73 μm. Over the course of the experiment, the two largest inclusions both expand by the same amount—to approximately four times their original size. Although this growth is probably not in the scale-free regime, the strains experienced around growing inclusions are much larger than the macroscopic failure strains (∼50%; see the Supplementary Materials), and it is likely that some chain breakage occurs. However, only the larger inclusion (with a final radius of 165 μm ≫ Γ/*E*) develops a crack tip, while the second inclusion (with a final radius 75 μm) does not. This is true in general: We find that large inclusions always develop into cracks, while small inclusions always stay rounded.

The cross-over point, Γ/*E*, is interesting, as this elasto-adhesive length is known to play an important role in soft fracture (*10*). In particular, it represents the effective process zone size at the tip of a large crack (*10*, *11*). Thus, our results can be interpreted physically, showing that scale-free growth is expected at scales much smaller than this characteristic process zone size.

This is completely analogous to metal cavitation. Voids in ductile metals expand when *P*_c_ ∼ σ*_y_*, where σ*_y_* is the yield stress (c.f. the Supplementary Materials) (*32*). Large cracks will also fail by brittle fracture when $P_{c}∼\sqrt{\Gamma E/w}$. Equating these, we find a crossover when $w∼\Gamma E/\sigma_{y}^{2}\equiv L_{p}$. This is a well-established transition length scale in ductile materials (*33*) and also the size of the plastic process zone for large cracks (*34*).

The main difference between the two types of material is in terms of scale. *L*_p_ in metals is typically macroscopic (e.g., *L*_p_ ∼ 1 cm in steel) (*10*). Thus, even macroscopic flaws in metals often grow in a ductile way, and void growth can be observed directly in experiments—for example, by postexamination of yielded samples. Hence, metals are commonly considered as ductile materials. However, in soft materials, Γ/*E* is microscopic (see Table 1). Thus, these soft materials also exhibit nonbrittle behavior, but this is more difficult to observe as it takes place at much smaller scales. By the time a cavity grows to a macroscopic size, it is in the brittle, crack-like regime where it will typically grow in a fast, unstable manner. Hence, elastomers are typically considered as having a brittle failure response.

## CONCLUSIONS

Every crack and cavity starts small. Thus, understanding their nucleation and early growth is crucial to understanding how they develop. Here, we have developed a method that allows us to grow and shrink microscopic cavities in soft materials with precise volume control, revealing the key physics underlying cavity growth in soft materials. We find that elastomers are not completely brittle materials, as commonly assumed. Instead, small, growing cavities appear to have much more in common with void growth in ductile metals, being accompanied by distributed damage around the surface of the cavity and growing at constant inflation pressure. This rationalizes a number of experimental observations, including measurements of “flaw-insensitive” rupture in soft materials (*35*). We hypothesize that this scale-free, inelastic behavior occurs in soft materials at scales smaller than the material length scale Γ/*E*—provided that surface tension effects are negligible (*23*).

The mechanism that we describe opens up many interesting directions for future work, including fundamental questions about the behavior of small flaws in soft materials. In particular, it will be important to develop further experimental and theoretical techniques to probe transitions between behaviors at different length scales. For example, we anticipate that one can extend cavitation techniques [e.g., (*3*, *14*)] to measure the critical cavitation pressure, *P*_c_, as a function of cavity size during growth and thus to fully explore the hypothesis in Fig. 4A.

Experiments like these could also be used to extract useful information about the large-strain behavior of materials. Normally, this is difficult to do with macroscopic experiments, as large samples break before they reach very high strains, but our approach allows us to stably induce very large strains without fracture. Thus, it could be possible to use measurements of *P*_c_ and the shapes of growing and shrinking droplets (like those in Figs. 2 and 3) to measure otherwise inaccessible material damage properties such as σ*_i_*. This will require a more detailed understanding of how damage occurs around a growing cavity, but this is seemingly an ideal topic for cutting-edge experimental techniques for imaging damage [e.g., (*36*)] and numerical simulations including stress softening or damage models. Ultimately, a knowledge of how materials fail at high strains will give us insight into the structure-property relationships that determine how materials fail, paving the way to allowing us to design novel, tough materials [e.g., (*37*–*39*)].

## MATERIALS AND METHODS

Our silicone gels consisted of a mixture of vinyl-terminated, silicone polymer chains (DMS-V31, Gelest) cross-linked with a methylhydrosiloxane–dimethylsiloxane copolymer (HMS-301, Gelest) with ratios of 69:1, 49:1, and 39:1 by mass (*22*). Respectively, these had *E* = 71,333 and 800 kPa. Cross-linking was achieved by mixing in a small amount of Karstedt’s catalyst (SIP6831.2, Gelest)—approximately 0.01% of the total mass of the sample. Once mixed, degassed, and poured into molds, samples were kept at 40°C for 24 hours to ensure complete cross-linking.

We measured *E* for the gels by indenting bulk samples (at least 10 mm in depth) with a 1-mm radius, cylindrical indenter on a texture analyzer with a 500-g load cell (TA.XTPlus, Stable Micro Systems). We assume sample incompressibility (a good assumption for soft gels and elastomers) and then extract *E* from the initial slope of the force-indentation curve [e.g., (*22*)].

We measured ϵ*_x_* in stretched samples using placing marks on the samples. ϵ*_x_* was then calculated by comparing the distance between marks during stretch and after subsequent stretch release.

We measured Γ using the Rivlin-Thomas pure shear test. Gel sheets (100-mm-wide, 2-mm-thick) were clamped between two long, straight clamps, with a distance of 20 mm between the clamps. We then extracted Γ by comparing the loading behavior of cracked and crack-free sheets, following (*38*) (see also the Supplementary Materials).

CARS microscopy was performed on a confocal Leica TCS SP8 microscope equipped with a tunable CARS laser (picoEmerald S, APE Berlin) and a nondescanned external detector (600 to 725 nm; Leica HyD). We used a 25× water immersion objective (Leica HC FLUOTAR L 25×/0.95 W VISIR). We visualized the silicone signal at a wave number of 2912 cm^−1^, using a 1032-nm Stokes beam and a 793.8-nm pump beam.

## Supplementary Material

aaz0418_Movie_S2.mov

aaz0418_Movie_S3.mp4

aaz0418_SM.pdf

aaz0418_Movie_S1.mov

## SUPPLEMENTARY MATERIALS

Supplementary material for this article is available at http://advances.sciencemag.org/cgi/content/full/6/13/eaaz0418/DC1 Section S1. Further experimental information Section S2. Numerical simulations Section S3. The pressure of a growing void in an elastic-plastic solid Fig. S1. Cyclical tensile testing of silicone samples. Fig. S2. The evolution of droplet image during growth in silicone gel with *E* = 71, 333 and 800 kPa and at three different applied strains. Fig. S3. CARS microscopy images of fluorinated oil droplets in silicone gels of different stiffnesses. Fig. S4. Results of a numerical model of cavity growth. Fig. S5. The evolution of the shape of a droplet in a neo-Hookean, incompressible solid, as the solid is uniaxially stretched. Fig. S6. A schematic showing the geometry used in calculating the critical pressure for growth in an elastic/perfectly plastic solid. Movie S1. Typical droplet growth in a stretched silicone gel. Movie S2. A close up of a single droplet growing in a stretched silicone gel. Movie S3. Droplets growing and shrinking in a stretched silicone gel.
