# Supplementary material for: Extreme cavity expansion in soft solids: Damage without fracture
Source: Sci Adv. 2020 Mar 27;6(13):eaaz0418. doi: 10.1126/sciadv.aaz0418 (PMC7101206; doi:10.1126/sciadv.aaz0418)
Supplement: aaz0418_SM.pdf [file aaz0418_SM.pdf]

Science Advances
AAAS

advances.sciencemag.org/cgi/content/full/6/13/eaaz0418/DC1

Supplementary Materials for

Extreme cavity expansion in soft solids: Damage without fracture

Jin Young Kim, Zezhou Liu, Byung Mook Weon, Tal Cohen, Chung-Yuen Hui, Eric R. Dufresne, Robert W. Style*

*Corresponding author. Email: robert.style@mat.ethz.ch

Published 27 March 2020, *Sci. Adv.* **6**, eaaz0418 (2020)
DOI: 10.1126/sciadv.aaz0418

The PDF file includes:

Section S1. Further experimental information
Section S2. Numerical simulations
Section S3. The pressure of a growing void in an elastic-plastic solid
Fig. S1. Cyclical tensile testing of silicone samples.
Fig. S2. The evolution of droplet image during growth in silicone gel with E = 71, 333 and 800 kPa and at three different applied strains.
Fig. S3. CARS microscopy images of fluorinated oil droplets in silicone gels of different stiffnesses.
Fig. S4. Results of a numerical model of cavity growth.
Fig. S5. The evolution of the shape of a droplet in a neo-Hookean, incompressible solid, as the solid is uniaxially stretched.
Fig. S6. A schematic showing the geometry used in calculating the critical pressure for growth in an elastic/perfectly plastic solid.

Other Supplementary Material for this manuscript includes the following:

(available at advances.sciencemag.org/cgi/content/full/6/13/eaaz0418/DC1)

Movie S1 (.mov format). Typical droplet growth in a stretched silicone gel.
Movie S2 (.mov format). A close up of a single droplet growing in a stretched silicone gel.
Movie S3 (.mp4 format). Droplets growing and shrinking in a stretched silicone gel.

Supplementary Material

Section S1. Further experimental information

A. Mechanical testing of silicone gels

We characterized the gel's mechanical properties E and Γ using indentation and fracture tests, with the results as given in the Table in the main paper. We measured E by indenting bulk gel samples (at least 10mm in depth) with a 1mm-radius, cylindrical indenter on a texture analyser with a 500g load cell (TA.XTPlus, Stable Microsystems). The initial slope of the force-indentation curve gives us $E/(1-\nu^2)$, where ν is the Poisson ratio. Then assuming sample incompressibility, so that $\nu = 1/2$ (a good assumption for soft gels and elastomers), we obtain E.

We measured Γ using the pure-shear test proposed by Rivlin and Thomas. 100mm wide, 2mm-thick sheets of silicone gel were clamped between two long, straight clamps, with a distance of 20mm between the clamps (see Figure 1f in the main paper). We measured the loading behavior of un-notched sheets, and sheets with an approximately 40mm long, seed crack, razor-cut from the sample edge along its centre-line. By observing the point at which the crack started to extend, we calculate Γ using the procedure described in [38].

Unfortunately, it is not easy to measure the stress at which inelasticity sets in, σ_i, as bulk test samples will typically fail (due to the growth of flaws) before the actual stress at which inelastic damage occurs is reached. However all the samples could be stretched by at least 50% before breaking, confirming that $\sigma_i \gtrsim E$.

B. Tensile testing

We looked for evidence of stress softening/a Mullins effect in our silicone materials by performing multiple sequential tensile tests of increasing magnitude on our silicone samples. An example showing the measured stress versus time for the silicone with $E = 333$kPa is shown in Figure S1a. When we plot this data on a stress/strain plot, all of the data collapses onto a single curve, as shown in Figure S1b. This is true for each of the different silicones that we use. The collapse shows that the silicone samples are highly elastic, and displays no stress softening/damage up to the point of sample failure.

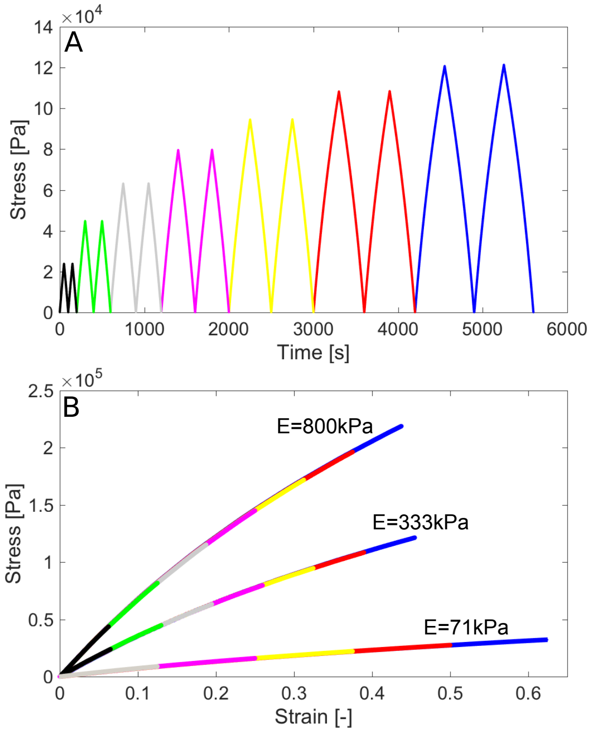

Fig. S1. Cyclical tensile testing of silicone samples. We created dogbone samples of each different material, and performed tensile tests of increasing amplitude on these until they failed (by breaking at the grips). A) The measured stress vs time for a sample with $E = 333$kPa to demonstrate the testing procedure. B) When the data from the tensile tests are plotted vs strain (calculated using the distance between grips), they collapse onto a single curve for each sample. This demonstrates that the samples are highly elastic and don't display any stress-softening).

C. Self-similar droplet growth

Figure S2 shows further examples of how the shapes of droplets evolve as they grow in stretched silicone gels

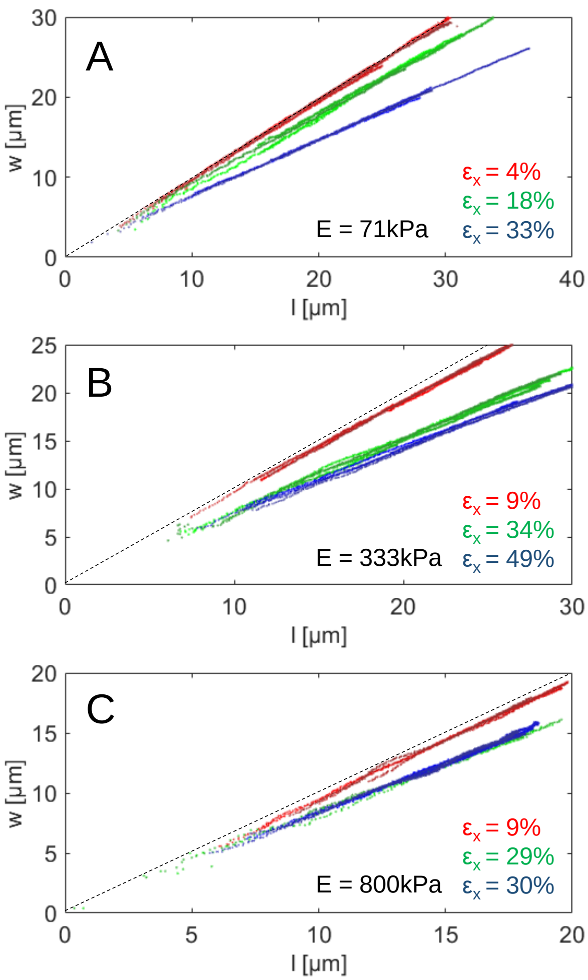

Fig. S2. The evolution of droplet image during growth in silicone gel with E = 71, 333, and 800 kPa and at three different applied strains. Each colour corresponds to one value of the applied strain. The different hues (5 for each set of conditions) correspond to different droplets. The dashed lines indicate an aspect ratio of 1.

of different stiffnesses. As with the plots in Figure 2a,b in the main paper, we see that droplets are always self-similar during growth.

D. CARS Microscopy

Using CARS microscopy, we measured the intensity of the silicone signal (with a Raman shift of 2912cm^{-1}) inside of pure fluorinated oil, and in droplets that grew inside silicone gels with $E = 71$ and 333kPa. A typical example of such a droplet with $E = 71$kPa is given in the Figure S3, along with an image of a fluorinated oil droplet inside uncrosslinked silicone for comparison.

The average signal intensity inside 19 droplets with $E = 71$kPa was 0.32 ± 0.2, while the average intensity inside 22 droplets with $E = 333$kPa was 0.4 ± 0.2. This could not be distinguished from the intensity inside

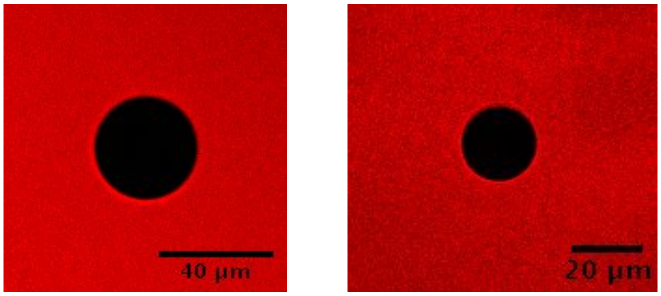

Uncrosslinked silicone E=71kPa

Fig. S3. CARS microscopy images of fluorinated oil droplets in silicone gels of different stiffnesses. Droplets of fluorinated oil in uncrosslinked silicone oil (left) and silicone gel with E = 71kPa (right).

droplets in the uncrosslinked silicone, which took a value of 0.26 ± 0.2. For reference, the average intensity of the silicone signal was approximately 22, and the standard deviation of the pixel noise in fluorinated oil was 0.35.

E. Videos

The three supplementary videos show different examples of droplets growing in stretched silicone gels. Video 1 shows growth in a silicone gel with $E = 333$kPa and $\epsilon_x = 49\%$. The scalebar is 100μm long. Video 2 shows a close up of one of the droplets in Video 1. Video 3 shows growth and shrinkage of droplets in a silicone gel with $E = 333$kPa and $\epsilon_x = 60\%$. In this video, the edge of the sample is at the top of the video, so evaporation occurs here, and droplets disappear first near this edge.

Section S2. Numerical simulations

The axisymmetric finite element (FE) model is shown schematically in Figure S4a, and implemented in a commercial finite element method (FEM) software, ABAQUS®. The initial droplet (blue region) is assumed to be a spherical cavity with radius R_0, which is surrounded by the silicone gel. The gel is modeled as an incompressible neo-Hookean solid with small-strain Young's modulus E, with strain-energy density function

$$W = \frac{E}{6}(I_1 - 3) \tag{1}$$

where I_1 is the trace of the right Cauchy-Green tensor.

The droplets are assumed to be sufficiently far apart so the gel outside occupies a region 100 times the radius R_0. A constant internal pressure P_c, is imposed on the deformed surface of the cavity. As in the experiments, we apply a strain ϵ_x in the axial x direction. Due to symmetry, only a quarter of the domain is modeled (see inset, Figure S4). On the axis of symmetry the radial displacement and shear traction are zero. The boundary on the top is traction free. The axial displacement and shear traction on the left are prescribed to be zero, and on

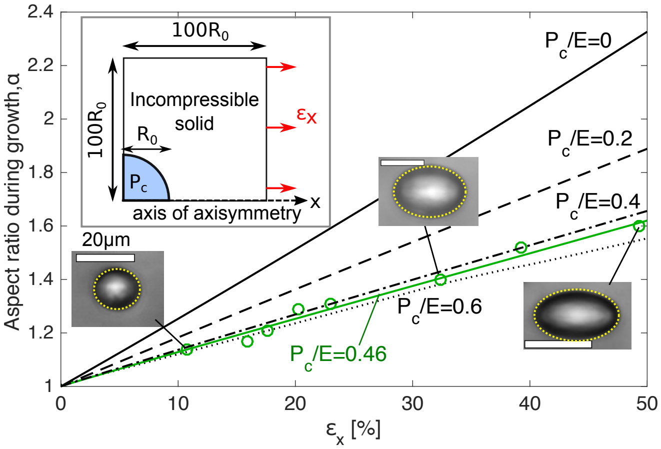

Fig. S4. Results of a numerical model of cavity growth. An inclusion is embedded inside an incompressible neo-Hookean solid with small-strain modulus E. The inclusion is then inflated to, and held at P_c while the solid is stretched with strain ϵ_x. At fixed P_c/E, α essentially is a linear function of ϵ_x. The green curve shows a fit to the $E = 71$kPa data (green circles). The inset images show the shapes of typical droplets at different stretch conditions, which are in excellent agreement with the fitted model.

the right boundary an axial displacement is imposed to give an average strain of ϵ_x. To balance the accuracy and efficiency of the computation, we choose a very fine mesh near the droplet's surface, while far away the element size increases rapidly. Hybrid axisymmetric elements CAX4H and CAX3H are used to simulate the incompressibility of the material. Our convergence test shows that further refinement of mesh does not affect the FE results.

As expected, the applied strain breaks symmetry and the cavity deforms into an oblate spheroid (e.g. Figure S5). In Figure S4, we show the aspect ratio of the deformed cavity as a function of the applied strain ϵ_x for different growth pressures on the cavity, alongside experimental data for the gel with $E = 71$kPa. These pressures P_c are normalized by the small-strain Young's modulus of the gel E. Interestingly, for a fixed pressure, we find a linear relationship between the aspect ratio and applied strain, consistent with experimental observation. Thus, it is possible to fit the experimental data – as demonstrated with the green line in the Figure, which corresponds to an inflation pressure $P_c/E = 0.46$.

Section S3. The pressure of a growing void in an elastic-plastic solid

Following Hill [32], we derive the critical pressure for self-similar growth of a pressurized void in an elastic/perfectly-plastic solid. We assume the geometry shown in Figure S6. A void of radius a_v is surrounded by a plastic shell of radius a_p that is embedded in an infinite elastic solid.

From Hill, the displacements in the radial direction at

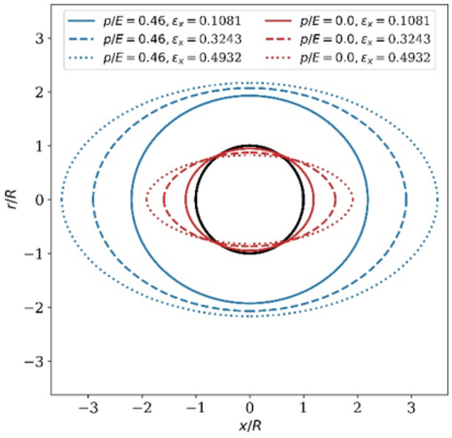

Fig. S5. The evolution of the shape of a droplet in a neo-Hookean, incompressible solid, as the solid is uniaxially stretched. The droplet is held at a fixed internal pressure, *P*. The different colors correspond to different inating pressures, while the black circle represents the initial size of the droplet.

the elastic-plastic boundary are given by

$$u(a_p) = \frac{\sigma_y a_p (1+\nu)}{3E} \tag{2}$$

where σ_y is the yield stress. Assuming that the void was originally of negligible size, and that elastic deformations are small, conservation of volume gives us that

$$\frac{4}{3}\pi a_v^3 = 4\pi a_p^2 u(a_p) \tag{3}$$

so

$$\frac{a_p}{a_v} = \left(\frac{E}{\sigma_y(1+\nu)}\right)^{1/3} \tag{4}$$

and we see that the growth is self-similar, with the plastic zone growing in proportion to the radius of the void.

To obtain the growth pressure of the void, we use the result from Hill that

$$P_c = 2\sigma_y \left(\log\left(\frac{a_p}{a_c}\right) + \frac{1}{3}\right) \tag{5}$$

so that

$$P_c = \frac{2\sigma_y}{3}\left(\log\left(\frac{E}{\sigma_y(1+\nu)}\right) + 1\right) \tag{6}$$

The logarithmic term will be $O(1)$ for typical materials, so we see that $P_c \sim \sigma_y$.

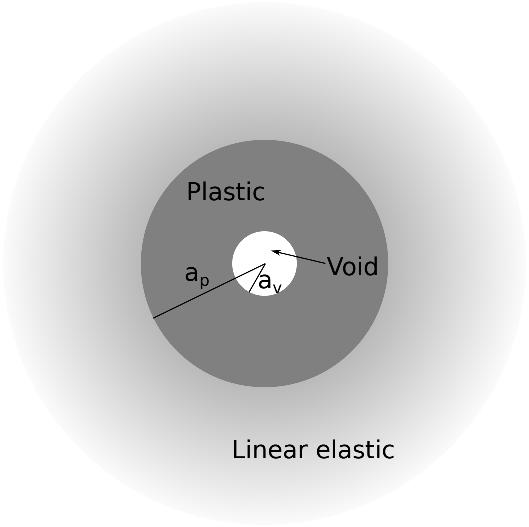

Fig. S6. A schematic showing the geometry used in calculating the critical pressure for growth in an elastic/perfectly plastic solid.
